# Draft Genome Sequence of Pseudomonas sp. Strains MWU12-2020 and MWU12-3103b, Isolated from Wild and Cultivated Cranberry Bogs in Massachusetts

**DOI:** 10.1128/mra.00568-22

**Published:** 2022-09-20

**Authors:** Andrea Ormsby, Scott Soby

**Affiliations:** a Biomedical Sciences, College of Graduate Studies, Midwestern University, Glendale, Arizona, USA; b College of Veterinary Medicine, Midwestern University, Glendale, Arizona, USA; SIPBS, University of Strathclyde

## Abstract

Here, we present the draft genome sequences of Pseudomonas sp. strains MWU12-2020 and MWU12-3103b, isolated from the rhizospheres of wild and cultivated cranberry bogs in southeastern Massachusetts; these strains are unrelated to known Pseudomonas species. The genomes of both isolates exceed 6 Mbp and contain predicted ice nucleation and type VI and III secretion system genes.

## ANNOUNCEMENT

Secondary metabolites produced by members of the genus Pseudomonas allow them to interact and survive within many different environments (1–5), but little is known about the role these bacteria play in soil dynamics or plant health in critical wetlands ecosystems. Pseudomonas sp. strain MWU12-2020 was isolated from wild cranberry bog soil (42.070624 N, 70.210548 W) and strain MWU12-3103b was isolated from cultivated cranberry bog roots (41.766767 N, 70.66842 W) during culture-dependent microbe surveys. Cranberry plants have a network of fine roots supported by ericoid mycorrhizae, which makes it difficult to differentiate between root and fungus and determine where the root zone is (6). In the case of MWU12-2020, there was no visible root or mycelium in the sample, but for MWU12-3103b, soil was attached to the root/mycelial network. The soil and rhizosphere samples were vortexed in sterile water; supernatants were plated onto King’s medium B (KMB) supplemented with 50 μg mL^−1^ each of ampicillin and cycloheximide and incubated at 26°C for 48 h. Fluorescent colonies were colony purified 3× on KMB and stored at −80°C in 34% glycerol. DNeasy blood and tissue kits (Qiagen, USA) were used to extract genomic DNA (gDNA) from overnight KMB broth cultures. Libraries for genomic sequencing were generated from enzymatically sheared DNA (≈500 bp) using a HyperPlus library preparation kit (Kapa Biosystems KK8514; Roche, USA). The sheared fragments were end repaired and A-tailed at the 3′ end. Indexed Illumina-compatible adapters (IDT number 00989130v2) were ligated to the A-tailed fragments; the library was cleaned using KAPA pure beads (KK8002) and then amplified using HiFi enzyme (KAPA KK2502). Fragment sizes were determined using an Agilent TapeStation device and then quantified using a KAPA quantitative PCR (qPCR) library quantification kit (KK4835) on a QuantStudio 5 system (Thermo Fisher, USA) for sequencing on the Illumina MiSeq platform (2 × 250-bp format). Using the Comprehensive Genome Analysis feature of PATRIC v3.6.12 (https://www.patricbrc.org) (7), assembly of the raw reads was completed using Unicycler v0.4.8 (8); the reads were polished using Pilon v1.23 (9), with default parameters except for enabling automated end trimming, and annotated using RASTtk (10). QUAST v5.0.2 and Trim Galore v0.4.0 were used within PATRIC (11, 12) for quality control and adapter trimming, respectively. A summary of the assembly and annotation is given in Table 1. Both isolates were placed in the genus Pseudomonas by genome BLAST distance phylogeny using TYGS v342 (13), but though most closely related to Pseudomonas koreensis DSM 16610^T^ (GenBank accession number JAAQYM000000000) (digital DNA-DNA hybridization [dDDH_d4_] = 51.2 and 43.7 for MWU12-2020 and MWU12-3103b, respectively), neither can be assigned to an existing species, and they do not belong in the same species (dDDH_d4_ = 51.2). Both genomes contain putative *inaA* genes for ice nucleation (14, 15) and type VI and III secretion systems (16–18), all of which have potential implications for interactions with other members of the ecosystem.

**TABLE 1:** Genomic data summary

Isolate	BioSample accession no.	GenBank accession no.	SRA accession no.	Genome size (bp)	No. of contigs	*N*_50_ (bp)	G+C content (%)	Mean read length (bp)	No. of reads	Coverage (×)	No. of CDSs	No. of rRNAs	No. of tRNAs
MWU12-2020	SAMN26879216	JALMEX000000000	SRR18531153	6,326,693	65	542,118	60.3	240.75	3,378,022	128	5,924	2	64
MWU12-3103b	SAMN26879228	JALMEY000000000	SRR18531152	6,275,820	55	276,792	60.2	236.97	3,012,488	114	5,831	2	64

### Data availability.

This whole-genome shotgun sequencing project has been deposited at DDBJ/EMBL/GenBank under accession numbers JALMEX010000000 and JALMEY010000000 and BioProject accession number PRJNA691338 (Table 1). The versions represented in the paper are the first versions. The RASTtk annotations are available under open license at Zenodo (https://zenodo.org/record/6412376#.Yxj1mEfMKUk).
